# Factors associated with breastfeeding difficulties in the early postpartum period in China: a cross-sectional study

**DOI:** 10.3389/fpubh.2026.1923736

**Published:** 2026-10-20

**Authors:** Fangfang Lei, Shurong Zhou, Yuanqiong Liu, Jie Xiang, Na Li, Yi Liu

**Affiliations:** 1 Department of Obstetric Nursing, West China Second University Hospital, Sichuan University, Chengdu, Sichuan, China; 2 Key Laboratory of Birth Defects and Related Diseases of Women and Children, Ministry of Education, Sichuan University, Chengdu, Sichuan, China

**Keywords:** associated factors, breastfeeding difficulties, early postpartum period, family support, postpartum depressive symptoms

## Abstract

**Objective:**

Within the unique clinical context of China, characterized by high cesarean section rates and specific postpartum practices, this study aims to investigate the prevalence and characteristics of breastfeeding difficulties in the early postpartum period (within 96 h postpartum) and identify associated factors, informing early clinical screening and targeted interventions.

**Methods:**

This cross-sectional study included 460 postpartum women recruited via convenience sampling from the obstetrics department of a tertiary specialized hospital in Chengdu between July 2023 and November 2024. All assessments were conducted between 24 and 96 h postpartum. Breastfeeding difficulty was operationally defined as the clinical presence of perceived insufficient milk, nipple/breast-related problems, or neonatal factors impeding effective feeding. Data were collected using a sociodemographic questionnaire, the Structured Clinical Breastfeeding Assessment Checklist, the LATCH scoring system, the Breastfeeding Self-Efficacy Scale-Short Form (BSES-SF), and the Edinburgh Postnatal Depression Scale (EPDS). Multivariate binary logistic regression was used to identify independently associated factors.

**Results:**

The prevalence of breastfeeding difficulties was 38.5% (177/460). The primary clinical manifestations were perceived insufficient milk supply (63.8%) and nipple-related problems (11.3%). Multivariate analysis demonstrated that infrequent breastfeeding (< 6 times/day; OR = 3.434), gestational diabetes mellitus (OR = 1.819), elevated pre-pregnancy BMI (24.0–27.9 kg/m^2^: OR = 2.484), and elevated postpartum depressive symptoms (OR = 3.365) were independently associated with higher odds of breastfeeding difficulties (all *P* < 0.05). Conversely, strong family support (OR= 0.911) and higher LATCH scores (OR = 0.646) showed a significant inverse association (all *P* < 0.05).

**Conclusion:**

Breastfeeding difficulties are common during the early postpartum period and are associated with a combination of maternal physiological conditions, feeding skills, and psychosocial factors. Elevated BMI, gestational diabetes, infrequent early sucking, and elevated postpartum depressive symptoms are key independently associated factors, whereas strong family support and good latching skills show a significant inverse association. Clinical support should prioritize early screening for high-risk women, integrating physical, skill-based, psychological, and familial support to overcome early lactation barriers.

## 1 Introduction

Breastfeeding remains a cornerstone of public health strategies for maternal and infant health. The World Health Organization (WHO) and the United Nations Children's Fund (UNICEF) strongly recommend exclusive breastfeeding for the first 6 months, as it provides optimal nutrition for early development (1, 2). It reduces the risk of infant respiratory and gastrointestinal infections and may lower the long-term incidence of type 2 diabetes and obesity (3–5). For mothers, it supports postpartum recovery and potentially mitigates the risk of chronic conditions like breast and ovarian cancers (6–8). Although the WHO aims to increase global exclusive breastfeeding rates to 60% by 2030 (1), current practices fall short. In China, shifting reproductive policies and a growing demand for evidence-based parenting highlight the need for supportive social and clinical environments (9). Yet, exclusive breastfeeding rates for infants under 6 months still lag behind national and WHO targets, with mothers facing persistent barriers across different stages of lactation (9). These barriers are deeply intertwined with the specific clinical environment in China. Routine clinical challenges, such as exceptionally high cesarean section rates, varied labor interventions, medically indicated maternal-infant separation, and poor adherence to early skin-to-skin contact, create a unique context that significantly disrupts the early onset of lactation (10, 11).

Early postpartum breastfeeding experiences are critical predictors of the success and duration of long-term feeding (12). These disruptions primarily affect secretory activation (SA), previously known as lactogenesis II. Occurring 36–96 h postpartum, SA marks the physiological shift from colostrum secretion to copious transitional milk production, characterized by breast fullness and a sudden increase in milk volume (13). This brief SA phase represents a critical window for breastfeeding difficulties, where initial disruptions can derail long-term success. Common challenges include delayed onset of lactation (DOL), breast engorgement, nipple pain, and perceived insufficient milk (13). DOL occurs in 20% to 44% of cases, a rate likely driven up by China's high cesarean delivery rates (12). Beyond physical discomfort, these early struggles are strongly associated with maternal anxiety and frustration, prompting premature and unnecessary formula supplementation (14). Early formula use reduces infant suckling, interfering with the mammary gland's autocrine feedback mechanisms and can predispose mothers to a cycle of dwindling milk supply (15–18). Consequently, mothers who struggle during SA and introduce formula early are significantly more likely to stop exclusive breastfeeding at 6 weeks and 6 months (19). Thus, SA-stage difficulties are not just short-term hurdles but key determinants of overall breastfeeding trajectories. Furthermore, while global health guidelines strongly advocate for continued breastfeeding up to 2 years of age or beyond to maximize immunological and developmental benefits, early lactation failure often permanently disrupts the trajectory toward these long-term milestones. Recognizing and mitigating early predictors of cessation is therefore not just about extending the initial duration of exclusive breastfeeding, but about empowering mothers to successfully navigate the transition to prolonged breastfeeding well past the first year of life.

While the general determinants of breastfeeding are well-documented, the specific factors associated with breastfeeding difficulties in the early postpartum period remain underexplored. Previous studies typically focus on broad variables like delivery mode or social support, rarely detailing how SA-phase difficulties shape long-term outcomes (20). For example, though DOL is linked to early cessation, direct evidence on how China's specific clinical context, which combines high cesarean rates with distinct obstetric practices, disrupts lactogenesis remains scarce (21). Because the assessment window of the present study (24–96 h postpartum) spans the period before and during conventional secretory activation, we define our outcome as breastfeeding difficulties in the early postpartum period rather than during secretory activation *per se*. To help address the gap described above, our study investigates the prevalence and multidimensional factors of breastfeeding difficulties in the early postpartum period (24–96 h postpartum). By identifying these early barriers, we aim to provide localized evidence for clinical interventions that help mitigate early breastfeeding cessation, ultimately supporting national policies to improve exclusive breastfeeding duration in China.

## 2 Methods

### 2.1 Study population

This study utilized a convenience sampling approach to recruit postpartum women from the obstetrics department of a Grade A tertiary specialized hospital in Chengdu between July 2023 and November 2024. The inclusion criteria were as follows: (1) aged ≥ 18 years; (2) intending to breastfeed or currently breastfeeding; (3) able to communicate and complete the questionnaire; (4) provided voluntary informed consent. The exclusion criteria were: (1) severe obstetric complications potentially affecting lactogenesis (e.g., severe preeclampsia, or massive postpartum hemorrhage with hemodynamic instability, requiring ICU admission, or requiring blood transfusion); (2) maternal medical contraindications to breastfeeding (e.g., human immunodeficiency virus, syphilis infection, or active tuberculosis); (3) neonatal congenital anomalies that physically preclude effective feeding (e.g., severe cleft lip or palate that prevents an effective latch); (4) personal decision not to breastfeed; (5) severe psychiatric or cognitive disorders precluding informed participation; (6) perinatal use of medications known to inhibit lactation (e.g., dopamine receptor agonists). This study protocol was approved by the Institutional Ethics Committee of the participating hospital.

### 2.2 Statistical analysis and sample size estimation

The occurrence of breastfeeding difficulties was treated as a dichotomous dependent variable. Therefore, a multivariate binary logistic regression model was employed to identify associated factors. Sample size requirements for the logistic regression were evaluated based on the “events per variable” (EPV) criterion. Traditional guidelines suggest a minimum of 10 positive events per estimated predictor parameter to ensure robust statistical estimates (22). Our revised, conceptually justified model contains 15 estimated predictor parameters. In this study, 177 of the 460 participants reported breastfeeding difficulties, yielding an actual EPV of 11.8 (177/15). This satisfies the traditional threshold, ensuring that the sample size is fully adequate for the current multivariate analysis.

### 2.3 Data collection instruments

#### 2.3.1 General information questionnaire

This questionnaire was developed by the research team based on an extensive review of domestic and international literature on breastfeeding, followed by expert consultation. It was designed to collect baseline characteristics, including: (1) Sociodemographic characteristics: age, height, weight, educational level, and occupation; (2) Clinical characteristics: gestational age at delivery, mode of delivery, gravidity, parity, history of abortion, pregnancy-related comorbidities and complications, intraoperative blood loss, use of a postoperative patient-controlled analgesia (PCA) pump, use of labor analgesia, presence of episiotomy or perineal laceration, postpartum hemorrhage, and history of breastfeeding experience and associated difficulties. Additionally, the questionnaire incorporated an investigator-developed 9-item Family Support Checklist. This formative index assessed specific family behaviors (e.g., providing postpartum meals, assisting with infant care, and offering verbal encouragement), with each item scored from 1 to 4, yielding a cumulative score ranging from 9 to 36. Higher scores indicate greater practical and emotional support from the family network. To ensure accuracy, objective medical and neonatal variables, including gestational age, mode of delivery, intraoperative blood loss, Apgar scores, neonatal congenital anomalies, and lingual frenulum status were not obtained via maternal self-report. Instead, they were extracted directly from the hospital's electronic medical records (EMR), including obstetric, anesthesia, and neonatal nursing documentation.

#### 2.3.2 Structured clinical breastfeeding assessment checklist

To standardize clinical observations, a Structured Clinical Breastfeeding Assessment Checklist was developed by the research team based on a comprehensive literature review and expert consensus. To ensure clinical rigor, its content validity was evaluated by senior lactation experts over two rounds. As a clinical inventory, this instrument standardizes the assessment of objective biological and behavioral parameters. It comprises four core domains: (1) Maternal physiological assessment (e.g., nipple morphology, breast pain); (2) Neonatal status assessment (e.g., neonatal oral anomalies, vital signs); (3) Breastfeeding process assessment (e.g., rooting reflex, sucking patterns); and (4) Maternal nutritional status assessment. The complete assessment checklist is provided in Supplementary File 2.

Operational Definition and Classification Algorithm of the Primary Outcome: in this study, “breastfeeding difficulties” was operationally defined as a dichotomous primary outcome (Yes/No). The classification was based on a combination of maternal self-report and objective clinical judgment by the IBCLC-certified nurses using the aforementioned scale. Specifically, a participant was classified into the “with breastfeeding difficulties” group if she met at least one of the following criteria during the assessment window: (1) Perceived insufficient milk supply causing significant maternal anxiety or leading to non-medically indicated formula supplementation; (2) Nipple or breast-related problems (e.g., severe pain disrupting feeding, visible nipple trauma, or severe breast engorgement); (3) Neonatal or interactive factors impeding effective feeding (e.g., infant inability to maintain a latch, severe crying at the breast, or poor weight gain requiring intervention). Participants who successfully breastfed without exhibiting any of these specific, documented clinical barriers were classified into the “without breastfeeding difficulties” group. This algorithm ensured a standardized and reproducible outcome assessment.

#### 2.3.3 The LATCH breastfeeding assessment tool

The LATCH assessment tool, developed by Jensen et al. (23), was used to evaluate breastfeeding effectiveness and maternal breastfeeding technique. This instrument comprises five dimensions: Latch, Audible swallowing, Type of nipple, Comfort (maternal), and Hold (positioning). Higher scores indicate superior breastfeeding skills and greater feeding effectiveness. The total score ranges from 0 to 10, with the following clinical interpretations: 8–10 points (good breastfeeding ability), 4–7 points (moderate ability), and 0–3 points (poor ability). The original Cronbach's α coefficient for this tool ranges from 0.87 to 0.95, indicating high internal consistency. Furthermore, this tool has been widely applied and validated in Chinese populations. Domestic clinical studies have demonstrated its good internal consistency (Cronbach's alpha =0.81) and excellent clinical applicability for evaluating postpartum breastfeeding effectiveness (24).

#### 2.3.4 Breastfeeding self-efficacy scale-short form (BSES-SF)

The BSES-SF, revised by Dennis (25), was employed to assess maternal confidence and internal beliefs regarding the maintenance of breastfeeding. The scale consists of 14 items, each rated on a 5-point Likert scale (ranging from 1, “not at all confident,” to 5, “always confident”). The total score ranges from 14 to 70, where higher scores reflect a higher level of breastfeeding self-efficacy. Lower scores are often considered an independent indicator for the premature cessation of breastfeeding in the early postpartum period. The Cronbach's α coefficient for this scale is 0.94, demonstrating excellent reliability. The Chinese version of the BSES-SF has been rigorously translated and validated in domestic populations, demonstrating excellent internal consistency (Cronbach's alpha = 0.927) and serving as a highly reliable tool for assessing maternal confidence in China (26).

#### 2.3.5 Edinburgh postnatal depression scale (EPDS)

EPDS developed by Cox et al. (27), was used to screen for and assess the severity of depressive symptoms in postpartum women. This instrument consists of 10 items, each rated on a 4-point Likert scale (0–3 points), resulting in a total score range of 0 to 30. Based on standard clinical screening criteria, a total score of ≥10 was established as the cutoff value for identifying elevated postpartum depressive symptoms (a positive screening result). Higher scores indicate more severe depressive symptoms. The Cronbach's α coefficient reported for this scale in the literature is 0.89. Furthermore, the Chinese version of the EPDS has been validated and widely applied in domestic populations, demonstrating good internal consistency (Cronbach's alpha = 0.76) and serving as a simple and highly reliable tool for the early screening of postpartum depressive symptoms in China (28).

### 2.4 Data collection and quality control

Data were collected by two clinical nurses from the obstetric department, both certified as International Board Certified Lactation Consultants (IBCLC). To ensure standardized assessment, these investigators completed rigorous training on the study protocol and scale administration prior to enrollment. Data collection methods were strictly stratified based on the nature of the variables. Subjective data (e.g., sociodemographic characteristics, previous breastfeeding experience) and psychometric scales (EPDS and BSES-SF) were self-reported by the mothers through questionnaire completion. Dynamic breastfeeding parameters—such as neonatal mental/behavioral status, rooting reflex, sucking and swallowing patterns, maternal breast engorgement, and nipple examinations—were determined through structured direct clinical observation and physical examination by the IBCLC-certified nurses during an active feeding session. For mothers who intended to breastfeed but had not yet successfully established feeding, these dynamic assessments were conducted during their active attempts to breastfeed (e.g., scoring 0 for the Latch and Audible swallowing domains if no latch was achieved). Similarly, their breastfeeding frequency was recorded based on the number of these attempted sessions at the breast. To consolidate this multidimensional data, investigators utilized a comprehensive Case Report Form as a centralized data extraction tool, combining the maternal self-reported questionnaires, objective EMR data, and clinical observations. Regarding quality control between the two assessors, an initial consensus-building pilot phase was conducted prior to formal data collection. Both IBCLC nurses underwent rigorous standardization training and jointly evaluated a pilot sample of 20 mother-infant dyads. Any discrepancies in clinical observation were discussed based on the assessment criteria until complete consensus was reached, ensuring high inter-rater standardization. During the formal study phase, each mother-infant dyad was assessed by only one of the standardized investigators to minimize participant burden. All clinical assessments and questionnaire administrations were conducted between 24 and 96 h postpartum (prior to hospital discharge). The exact timing of assessment was recorded for each participant. For mothers assessed on postpartum day 1 (24–36 h), early signs of feeding difficulties, such as latching issues and perceived insufficient colostrum, were evaluated as early barriers preceding full secretory activation. During the survey, investigators explained the study's objectives and confidentiality protocols to participants and their families. Upon obtaining written informed consent, questionnaires were administered face-to-face. For participants unable to complete the forms independently due to physical discomfort, investigators read the items aloud in a neutral manner and recorded responses verbatim to prevent leading cues. Questionnaires were reviewed on-site for completeness; any missing items were addressed immediately with the participant's consent. Data entry was conducted using a double-verification mechanism to ensure dataset integrity and minimize input errors.

### 2.5 Statistical analysis

Data were analyzed using SPSS 27.0. Regarding missing data, a complete case analysis approach was employed. Because questionnaires were reviewed on-site and 5 participants with unrecoverable incomplete data were excluded prior to analysis, there were no missing values in the final dataset of 460 participants, and therefore no imputation methods were required. Categorical data were expressed as frequencies and percentages, and compared using the chi-square test. The normality of continuous variables was assessed using the Kolmogorov-Smirnov test. Because variables such as breastfeeding self-efficacy, family support, EPDS scores, and LATCH scores exhibited non-normal distributions, they were presented as medians with interquartile ranges (IQR) and compared between groups using the non-parametric Mann–Whitney U test. Categorical variables were expressed as frequencies and percentages, and compared using the Pearson chi-square (*X*^2^) test or Fisher's exact test, as appropriate. For categorical variables with more than two levels, the reported *P*-values represent the overall test of association. *Post hoc* pairwise comparisons were not performed at the univariate stage, as the primary purpose of this analysis was to screen candidate variables. Instead, specific comparisons between groups were evaluated within the multivariable logistic regression model, where individual categories were directly compared against a designated reference group. To identify independent factors associated with breastfeeding difficulties, a multivariate binary logistic regression model was constructed. Variables demonstrating statistical significance in the univariate analysis (*P* < 0.05), along with clinically established *a priori* confounders (e.g., maternal age, mode of delivery, and parity), were included in the multivariable model to rigorously adjust for potential confounding. Findings were reported as odds ratios (OR) with 95% confidence intervals (95% CI). Prior to multivariable modeling, multicollinearity among the independent variables was assessed using the Variance Inflation Factor (VIF). Variables with a VIF ≥ 5 were considered to indicate severe collinearity and were excluded from the model to avoid double counting. Furthermore, to address potential criterion contamination between the LATCH score and the operational definition of breastfeeding difficulties, a sensitivity analysis was conducted. We excluded the total LATCH score from the multivariable logistic regression model to evaluate the robustness of the remaining predictors. Model fit was evaluated using the Hosmer–Lemeshow goodness-of-fit test, Nagelkerke's pseudo-*R*^2^, and the Area Under the Receiver Operating Characteristic Curve. The assumption of linearity in the logit for continuous predictors was assessed using the Box–Tidwell test. All tests were two-sided, with *P* < 0.05 considered statistically significant.

## 3 Results

### 3.1 General characteristics of the study population

A total of 500 postpartum women were initially screened. Based on the predefined criteria, 25 women were excluded due to ineligibility (e.g., medical contraindications). Of the remaining 475 eligible women, 10 refused participation (or made a personal decision not to breastfeed), and 5 were excluded due to incomplete questionnaire data. The detailed screening and enrollment process is illustrated in Figure 1. Ultimately, 460 women were included in the final analysis, of which 177 (38.5%) reported experiencing breastfeeding difficulties during the early postpartum stage. As shown in Table 1, when classified by their primary clinical manifestation, the most frequently reported difficulty was perceived insufficient milk supply (63.8%), followed by nipple-related problems (11.3%) and neonatal health issues (6.8%). Detailed mutually exclusive classifications of these primary difficulties are presented in Table 1.

**Figure 1:**
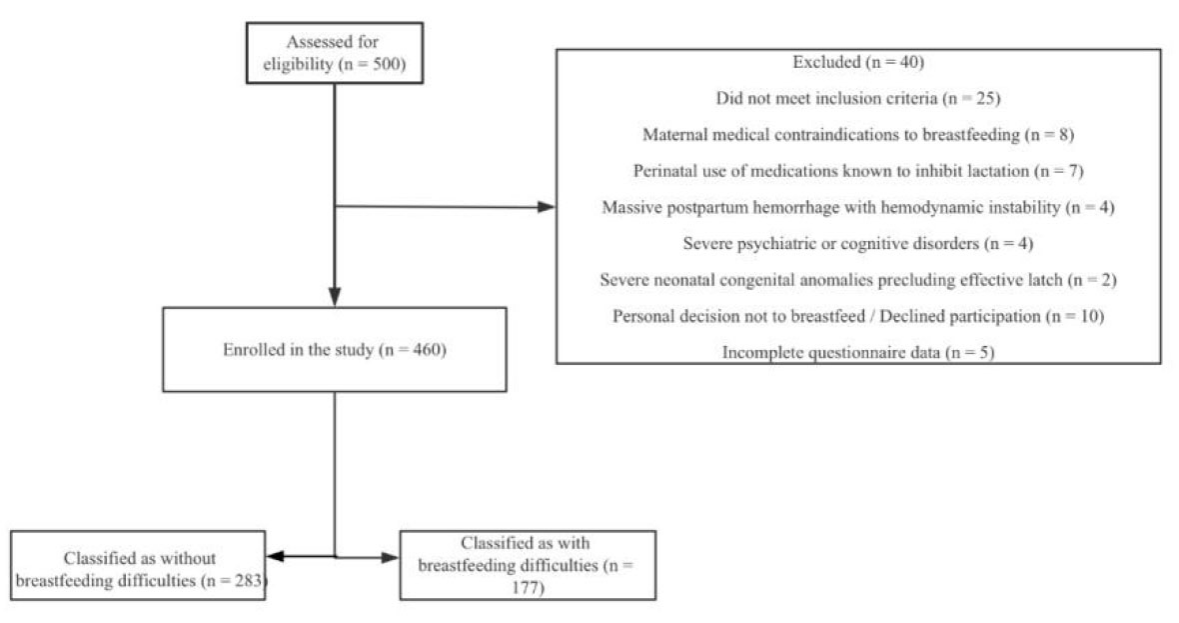
Participant flow diagram outlining the screening, exclusion, and enrollment process according to predefined eligibility criteria.

**Table 1:** Classification of breastfeeding difficulties in the early postpartum period (*n* = 177).

Classification of breastfeeding difficulties	*N* (%)
Primary difficulties	
Perceived insufficient milk supply	113 (63.8%)
Nipple-related problems	20 (11.3%)
Secondary/Other issues	
Neonatal health issues (e.g., twins, jaundice, protein allergy, lactose intolerance, history of hematochezia)	12 (6.8%)
Insufficient infant satiety/Poor weight gain	11 (6.2%)
Breast-related problems	7 (4.0%)
Insufficient family support	5 (2.8%)
Returning to work	5 (2.8%)
Perceived inadequate breast milk nutrition	4 (2.3%)

Data are *n* (%). Percentages calculated from total symptomatic participants (*n* = 177). Each participant was assigned to a single mutually exclusive category representing their primary clinical difficulty.

### 3.2 Univariate analysis of breastfeeding difficulties in the early postpartum period

The core baseline characteristics and univariate analysis results of the study population are presented in Table 2. Additionally, the detailed clinical observations regarding maternal breast conditions, neonatal status, and specific feeding behaviors have been provided in Supplementary Table S1. Significant differences were observed between the two groups (those with and without breastfeeding difficulties) regarding maternal age, body mass index (BMI), mode of delivery, parity, gestational hypertension, gestational diabetes mellitus (GDM), nipple morphology, daily breastfeeding frequency, LATCH scores, breastfeeding self-efficacy, family support, and elevated postpartum depressive symptoms (all *P* < 0.05). The EPDS total score ranged from 5 to 23, and 52 women (11.3%) screened positive (EPDS ≥ 10). Although the continuous EPDS total score did not differ significantly between groups (*P* = 0.209), the proportion screening positive was higher among women with breastfeeding difficulties (20.9% vs. 5.3%).

**Table 2:** Univariate analysis of core demographic characteristics and factors associated with breastfeeding difficulties in the early postpartum period [*n* (%)] (*n* = 460).

Characteristics	Without breastfeeding difficulties (*n* = 283)	With breastfeeding difficulties (*n* = 177)	Test statistic	*P*
Breastfeeding self-efficacy [Median (IQR)]	44 (39, 49)	43 (29, 30)	2.445	0.014
Family support [Median (IQR)]	24 (21, 27.5)	23 (21, 25)	3.815	< 0.001
EPDS total scores [Median (IQR)]	7 (6, 8)	7 (6, 9)	1.257	0.209
LATCH score [Median (IQR)]	7 (6, 9)	5 (4, 6)	9.062	< 0.001
Age (years)				
< 35	180 (63.6%)	92 (52.0%)	6.091	0.014
≥35	103 (36.4%)	85 (48.02%)		
Gestational age at delivery (weeks)				
< 34	11 (3.89%)	8 (4.52%)	0.213	0.899
34–36^+6^	30 (10.6%)	17 (9.6%)		
≥37	242 (85.51%)	152 (85.88%)		
Mode of delivery				
Vaginal delivery	199 (70.3%)	100 (56.5%)	9.143	0.002
Cesarean section	84 (29.68%)	77 (43.5%)		
Educational level				
Primary school or below	32 (11.31%)	25 (14.12%)	1.238	0.744
Junior high school	61 (21.55%)	35 (19.77%)		
High school/College	112 (39.58%)	73 (41.24%)		
Bachelor's degree or above	78 (27.56%)	44 (24.86%)		
Occupation				
Employed	240 (84.81%)	155 (87.57%)	0.686	0.407
Unemployed	43 (15.19%)	22 (12.43%)		
Gravidity				
1	142 (50.18%)	94 (53.11%)	2.469	0.291
2	94 (33.22%)	63 (35.59%)		
≥3	47 (16.61%)	20 (11.3%)		
Parity				
1	203 (71.73%)	155 (87.57%)	16.084	< 0.001
2	49 (17.31%)	15 (8.47%)		
≥3	31 (10.95%)	7 (3.95%)		
Daily breastfeeding frequency				
8–12 times/day	205 (72.44%)	100 (56.5%)	18.564	< 0.001
< 6 times/day	66 (23.32%)	74 (41.81%)		
6–7 times/day	12 (4.24%)	3 (1.69%)		
Hypertensive disorders of pregnancy				
No	245 (86.57%)	138 (77.97%)	5.787	0.016
Yes	38 (13.43%)	39 (22.03%)		
GDM				
No	229 (80.92%)	127 (71.75%)	5.23	0.022
Yes	54 (19.08%)	50 (28.25%)		
Complicated by heart disease				
No	269 (95.05%)	170 (96.05%)	0.246	0.62
Yes	14 (4.95%)	7 (3.95%)		
Complicated by anemia				
No	263 (92.93%)	170 (96.05%)	1.909	0.167
Yes	20 (7.07%)	7 (3.95%)		
Intraoperative blood loss during cesarean section (*n* = 161)				
< 1000 ml	62 (73.81%)	65 (84.42%)	2.712	0.100
≥1000 ml	22 (26.19%)	12 (15.58%)		
Use of PCA pump				
Yes	174 (61.48%)	99 (55.93%)	1.391	0.238
No	109 (38.52%)	78 (44.07%)		
Labor analgesia				
Yes	134 (47.35%)	84 (47.46%)	0.001	0.982
No	149 (52.65%)	93 (52.54%)		
Episiotomy				
Yes	86 (30.39%)	60 (33.9%)	0.619	0.431
No	197 (69.61%)	117 (66.1%)		
Perineal tear				
Yes	50 (17.67%)	35 (19.77%)	0.321	0.571
No	233 (82.33%)	142 (80.23%)		
Estimated blood loss (vaginal delivery) (*n* = 299)				
< 500 ml	154 (77.39%)	74 (74.00%)	0.439	0.507
≥500 ml	45 (22.61%)	26 (26.00%)		
Previous breastfeeding experience				
No	246 (86.93%)	162 (91.53%)	2.298	0.13
Yes	37 (13.07%)	15 (8.47%)		
Previous breastfeeding problems				
No	244 (86.22%)	146 (82.49%)	1.176	0.278
Yes	39 (13.78%)	31 (17.51%)		
Time of assessment (hours postpartum)				
24–47 h	101 (35.69%)	70 (39.55%)	0.79	0.674
48–71 h	85 (30.04%)	52 (29.38%)		
≥72 h	97 (34.28%)	55 (31.07%)		
Nipple type				
Everted	243 (85.87%)	84 (47.46%)	91.328	< 0.001
Flat	21 (7.42%)	18 (10.17%)		
Inverted	19 (6.71%)	75 (42.37%)		
BMI classification				
< 18.5	49 (17.31%)	21 (11.86%)	8.336	0.04
18.5–23.9	189 (66.78%)	114 (64.41%)		
24–27.9	40 (14.13%)	32 (18.08%)		
≥28	5 (1.77%)	10 (5.65%)		
Elevated depressive symptoms				
No	268 (94.7%)	140 (79.1%)	26.443	< 0.001
Yes	15 (5.3%)	37 (20.9%)		

Test statistics represent *Z* values derived from the Mann–Whitney *U* test for non-normally distributed continuous variables, and χ^2^ values for categorical variables, unless explicitly marked as Fisher's exact test. For categorical variables with more than two levels, the reported *P* value represents the overall test across all categories.

### 3.3 Multivariable logistic regression analysis of breastfeeding difficulties

The occurrence of breastfeeding difficulties during the early postpartum stage was treated as the dependent variable (0 = no difficulty, 1 = presence of difficulty). Variables identified as statistically significant in the univariate analysis were considered for the multivariable binary logistic regression model. The definition and coding of these independent variables are summarized in Table 3. To prevent tautological association and criterion contamination, isolated anatomical predictors (e.g., nipple morphology) were explicitly excluded from the final multivariable model, as this construct is already evaluated within the “Type of nipple” dimension of the LATCH tool. After adjusting for potential confounding factors (including maternal age, mode of delivery, and parity) and confirming the absence of multicollinearity (the maximum VIF value was 1.86, well below the threshold of 5), the results indicated that lower daily breastfeeding frequency, GDM, elevated BMI, and elevated postpartum depressive symptoms were independently associated with higher odds of breastfeeding difficulties (OR > 1.00, *P* < 0.05). Conversely, strong family support and higher LATCH scores were identified as having an independent inverse association with breastfeeding difficulties during this period (OR < 1, *P* < 0.05). Age, mode of delivery, parity, hypertensive disorders of pregnancy, and breastfeeding self-efficacy did not show a statistically significant independent association with breastfeeding difficulties in the final multivariable model (*P* > 0.05). The detailed statistical parameters are presented in Table 4, and the adjusted Odds Ratios with their 95% confidence intervals are visually summarized in the accompanying forest plot (Figure 2). To provide a clinically meaningful interpretation for the continuous variables, we calculated the adjusted odds ratios for specific clinical increments. For the Family Support checklist, a 5-point higher Family Support score was associated with 37.3% lower odds of early breastfeeding difficulties (OR = 0.627, 95% CI: 0.473–0.837). For the LATCH tool, where the total score ranges from 0 to 10, a clinically relevant 2-point higher LATCH score was associated with 58.3% lower odds of feeding barriers (OR = 0.417, 95% CI: 0.327–0.531). The BSES-SF score demonstrated a trend toward lower odds of breastfeeding difficulties, where a 5-point higher score was associated with an OR of 0.868 (95% CI: 0.734–1.025). The primary multivariable model demonstrated good fit (Hosmer–Lemeshow *P* = 0.227, Nagelkerke *R*^2^ = 0.411, AUC = 0.829). Furthermore, the sensitivity analysis excluding the LATCH score confirmed the robustness of our main findings. This unconstrained sensitivity model also exhibited an adequate fit (Hosmer–Lemeshow *P* = 0.983, Nagelkerke *R*^2^ = 0.280, AUC = 0.764). As detailed in Supplementary Table S2, even without the LATCH score, lower breastfeeding frequency, elevated BMI, GDM, and elevated postpartum depressive symptoms remained independently associated with increased odds of breastfeeding difficulties. Regarding continuous predictors, although the Box–Tidwell test indicated slight deviations from strict log-linearity, they were retained as continuous variables to preserve standardized clinical interpretability.

**Table 3:** Definition and coding of independent variables.

Variables	Coding
Age	1 = < 35 years, 2 = ≥35 years
Parity	1 = 1, 2 = 2, 3 = ≥3
Mode of delivery	1 = Vaginal delivery, 2 = Cesarean section
Daily breastfeeding frequency	1 = 8–12 times, 2 = < 6 times, 3 = 6–7times
Hypertensive disorders of pregnancy	0 = No, 1 = Y es
Gestational diabetes mellitus	0 = No, 1 = Yes
Nipple morphology	1 = Inverted, 2 = Flat, 3 = Everted (after stimulation)
BMI	1 = < 18.5, 2 = 18.5–23.9, 3 = 24.0–27.9, 4 = ≥28.0
Depressive symptoms screening	1 = Negative (score < 10), 2 = Positive (score ≥10)
Breastfeeding self-efficacy	Raw score
Family support	Raw score
LATCH score	Raw score

**Table 4:** Multivariable logistic regression analysis of factors associated with breastfeeding difficulties in the early postpartum period (*n* = 460).

Variables	β	SE	Wald *χ^2^*	*P* value	OR (95% CI)
(Intercept)	6.378	1.250	26.059	< 0.001	589.021 (50.881–6,818.831)
Breastfeeding self-efficacy	−0.028	0.017	2.757	0.097	0.972 (0.940–1.005)
Family support	−0.093	0.029	10.245	0.001	0.911 (0.861–0.965)
LATCH score	−0.437	0.062	49.942	< 0.001	0.646 (0.572–0.729)
Age					
< 35 years	–	–	–	–	1.00 (Ref.)
≥35 years	0.390	0.249	2.450	0.118	1.477 (0.906–2.407)
Mode of delivery					
Vaginal delivery	–	–	–	–	1.00 (Ref.)
Cesarean section	0.402	0.243	2.729	0.099	1.494 (0.928–2.407)
Parity					
1 time	–	–	–	–	1.00 (Ref.)
2 times	−0.737	0.417	3.128	0.077	0.479 (0.212–1.083)
≥3 times	−0.456	0.522	0.762	0.383	0.634 (0.228–1.763)
Daily breastfeeding frequency					
8–12 times/day	–	–	–	–	1.00 (Ref.)
< 6 times/day	1.234	0.261	22.384	< 0.001	3.434 (2.060–5.725)
6–7 times/day	−0.242	0.840	0.083	0.773	0.785 (0.151–4.075)
Hypertensive disorders of pregnancy					
No	–	–	–	–	1.00 (Ref.)
Yes	0.534	0.321	2.774	0.096	1.706 (0.910–3.201)
Gestational diabetes mellitus					
No	–	–	–	–	1.00 (Ref.)
Yes	0.598	0.284	4.427	0.035	1.819 (1.042–3.177)
BMI classification					
< 18.5	–	–	–	–	1.00 (Ref.)
18.5–23.9	0.418	0.345	1.473	0.225	1.519 (0.773–2.986)
24–27.9	0.910	0.419	4.716	0.030	2.484 (1.093–5.649)
≥28	1.245	0.765	2.649	0.104	3.471 (0.776–15.540)
Elevated depressive symptoms					
No	–	–	–	–	1.00 (Ref.)
Yes	1.213	0.397	9.330	0.002	3.365 (1.545–7.329)

The multivariable logistic regression model was adjusted for demographic and clinical confounding factors, specifically maternal age, mode of delivery, and parity. For continuous scales (Breastfeeding self-efficacy, Family support, and LATCH score), the ORs presented in this table represent a 1-point increase. Translations of these effects into clinically meaningful increments (e.g., 2-point or 5-point increases) are detailed in the text.

**Figure 2:**
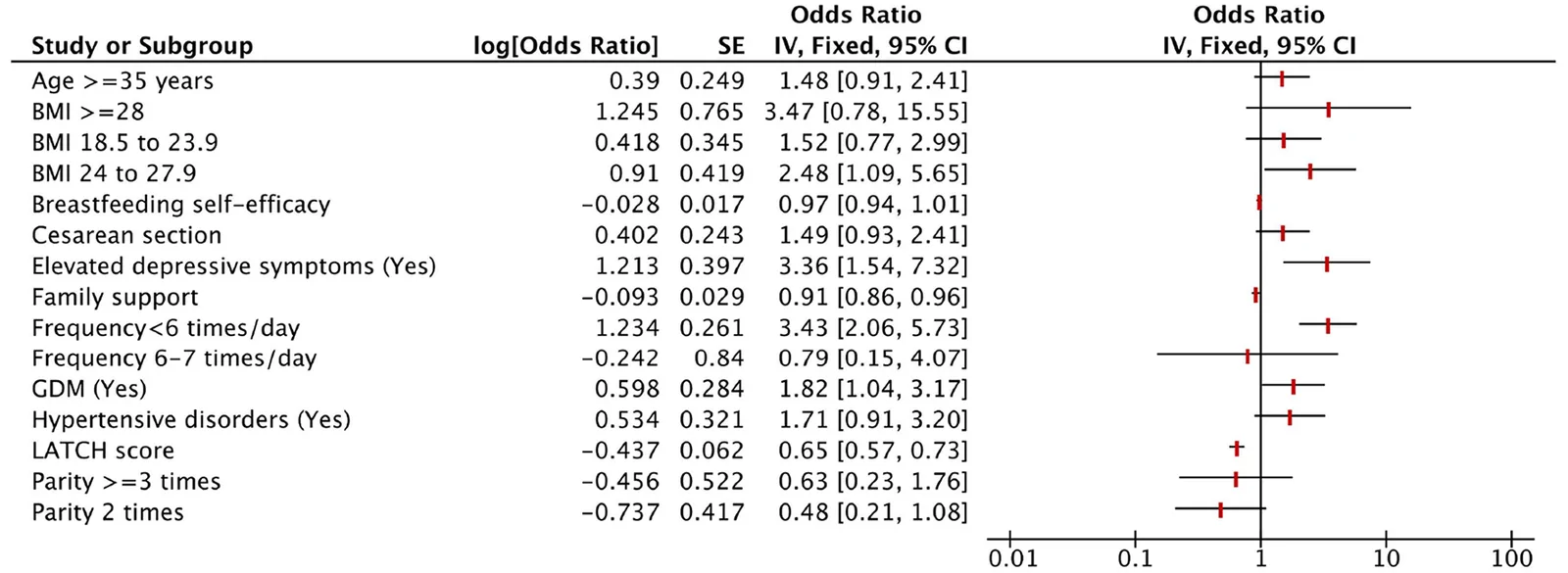
Forest plot illustrating the adjusted odds ratios and 95% confidence intervals for factors associated with early postpartum breastfeeding difficulties.

## 4 Discussion

### 4.1 Current status of breastfeeding difficulties in the early postpartum period requires improvement

Our findings indicate that out of 460 participants, 177 (38.48%) experienced breastfeeding difficulties in the early postpartum period, which falls within the range of 20%−44% reported in previous studies, suggesting that the lactation initiation performance of our sample is consistent with common clinical observations (31, 32). Secretory activation represents a critical physiological window for the establishment of breastfeeding behavior. Failure to identify and address difficulties during this period may increase the risks of non-medically indicated formula supplementation and premature cessation of breastfeeding (33). Further analysis of these difficulties revealed that perceived insufficient milk (63.8%) and nipple-related issues (11.3%) were the primary clinical manifestations. Existing research indicates that primary physiological lactation insufficiency in early secretory activation is relatively rare; instead, it is frequently characterized as perceived insufficient milk, which is often associated with maternal misconceptions regarding neonatal gastric capacity, anxiety concerning physiological weight loss, and postpartum fatigue (34). Furthermore, nipple pain or fissuring suggests potential obstacles in maternal-infant latching techniques. If left unaddressed, the physiological stress induced by pain may partially inhibit oxytocin release, thereby impairing the milk-ejection reflex and milk drainage, which creates a negative physiological feedback loop (35, 36). In summary, breastfeeding difficulties in the early postpartum period are multifactorial. Based on this, clinical practice should prioritize shifting the focus of breastfeeding support to earlier stages. Healthcare providers should establish routine assessment mechanisms to identify high-risk populations, thereby providing a clinical entry point for subsequent refined interventions.

### 4.2 Association of physiological and pathological factors with breastfeeding difficulties in the early postpartum period

Our findings highlight that specific maternal physiological and pathological conditions, notably an elevated BMI (≥24) and GDM, were significantly and independently associated with higher odds of breastfeeding difficulties in the early postpartum period. Elevated postpartum depressive symptoms are also independently associated with higher odds of these difficulties. In contrast, mothers equipped with strong family backing and better latching skills (reflected in higher LATCH scores) showed a significant inverse association with these early feeding challenges.

These metabolic and structural constraints may disrupt the critical endocrinological shifts required for secretory activation. For example, the insulin resistance characteristic of GDM can directly blunt the autocrine regulation of prolactin within the mammary alveoli (37, 38). Similarly, maternal overweight or obesity often delays the prolactin surge, severely complicating the timely onset of secretory activation. Given this evidence, relying on one-size-fits-all antenatal breastfeeding education is insufficient. Postpartum care should instead pivot to targeted, risk-stratified interventions. By establishing standardized early risk screenings, multidisciplinary teams can immediately step in with hands-on, non-pharmacological support, such as tailored postural adjustments for flat/inverted nipples or early manual milk expression. This proactive clinical approach warrants prospective evaluation to support early lactation trajectories in high-risk mothers (36, 39).

### 4.3 The inverse association of standardized breastfeeding behaviors and skills with breastfeeding difficulties in the early postpartum period

Beyond maternal physiological conditions, proficient feeding behaviors show a crucial inverse association in the early postpartum period. Notably, mothers who breastfeed fewer than six times a day have markedly higher odds of early difficulties compared to those meeting the recommended 8–12 sessions. Early postpartum milk synthesis relies heavily on local “supply-and-demand” autocrine regulation (40). High-frequency, effective suckling ensures timely milk removal, preventing the accumulation of feedback inhibitors of lactation within the mammary alveoli and sustaining prolactin receptor sensitivity (29). When feeding frequency drops, delayed milk emptying may disrupt this cycle, which is consistent with the high rates of “perceived insufficient milk” observed in our cohort (41). Technical proficiency is equally critical (42). A LATCH score that is 2 points higher, such as moving from moderate to good feeding ability, is robustly associated with approximately 58% lower odds of feeding barriers. A deep, standardized latch is physiologically expected to provide two immediate benefits: it maximizes milk transfer efficiency to drive continued lactogenesis, and it significantly minimizes mechanical friction on the nipple (43). By mitigating acute nipple pain, mothers may avoid the stress-induced inhibition of the hypothalamic milk-ejection reflex, which may help sustain early lactation. Therefore, preventing early feeding barriers may require clinical adherence to “early skin-to-skin contact and early suckling” protocols (44). Healthcare providers must move beyond passive observation, using standardized tools like LATCH to dynamically assess and correct feeding techniques. Actively assisting mothers in maintaining an on-demand feeding schedule may provide mechanical stimulation that helps mitigate technical barriers and premature weaning.

### 4.4 The interrelated association of psychosocial factors with breastfeeding difficulties in the early postpartum period

Psychosocial wellbeing, maternal self-efficacy, and family support form an interrelated network that is closely associated with breastfeeding difficulties in the early postpartum period. Our data show that elevated postpartum depressive symptoms are independently associated with three-fold higher odds of breastfeeding difficulties (OR = 3.365). This clinical finding aligns with the neuroendocrinological understanding that stress and depression activate the hypothalamic-pituitary-adrenal axis; the resulting cortisol fluctuations may suppress oxytocin release, thereby inhibiting the milk-ejection reflex (45). Beyond hormonal disruption, low mood may blunt a mother's proactive coping strategies, potentially derailing the consistent implementation of on-demand feeding. Internally, maternal confidence plays a nuanced role. While higher breastfeeding self-efficacy demonstrated a trend toward an inverse association, it did not remain an independent significant factor in the fully adjusted multivariable model (OR = 0.972, *P* = 0.097). However, as shown in our sensitivity analysis, its significant inverse association (OR = 0.966) becomes apparent when LATCH scores are excluded. Mothers with strong self-efficacy generally exhibit greater psychological resilience when confronting acute challenges like physiological engorgement, nipple pain, or perceived low milk supply (46). It is important to acknowledge that timely clinical interventions, including medically indicated formula supplementation, are sometimes mandated to ensure infant health, such as in cases of continuous weight loss. In these complex situations, strong maternal confidence helps mothers navigate these necessary interventions more adaptively without viewing them as permanent failures.

Externally, a robust family support system may act as a buffer. Our multivariable model indicates that every 5-point higher score on the practical family support checklist was associated with 37% lower odds of early feeding difficulties. In the specific context of Chinese maternity wards, hospital regulations generally encourage 24-h maternal-infant rooming-in and permit one designated family member to provide round-the-clock bedside accompaniment. With this continuous physical presence, practical assistance from primary caregivers, such as infant burping and dietary care, may mitigate maternal physical exhaustion (47). Concurrently, their continuous emotional validation may alleviate maternal anxiety and feelings of helplessness in the early postpartum period (30). Crucially, these psychosocial domains do not operate in isolation. A stable family environment may be associated with better maternal mood and higher self-efficacy, which may provide a psychological foundation for successful early lactation.

Therefore, modern obstetric care must integrate emotional screening into routine postpartum clinical pathways. Identifying mothers with elevated depressive symptoms or low self-efficacy early allows for immediate, targeted psychological support. Moreover, nursing education must expand its focus beyond the mother to include primary caregivers. Establishing a “clinician-nurse-patient-family” collaborative model may help ensure that caregivers are equipped to provide both the substantive and emotional support necessary to safeguard early lactation.

## 5 Conclusion

Breastfeeding difficulties in the early postpartum period are prevalent among postpartum women. This clinical phenomenon is rarely associated with a single factor but rather with multidimensional interactions between maternal physiological and pathological status, breastfeeding skills, and psychosocial support networks. Multivariate analysis indicates that elevated pre-pregnancy BMI (24.0–27.9), GDM, low frequency of early infant sucking (< 6 times/day), and elevated postpartum depressive symptoms are independently associated with higher odds of feeding difficulties during this critical period. Conversely, standardized latching techniques (indicated by higher LATCH scores) and strong core family support show significant inverse associations. These findings provide evidence-based insights for clinical nursing practice. It is recommended that healthcare providers shift the focus of interventions toward earlier stages by implementing screening and dynamic assessments for high-risk populations. During the early postpartum period, efforts should move beyond providing guidance on high-frequency, standardized breastfeeding techniques; equal emphasis must be placed on maternal psychological status, and primary caregivers should be integrated into a collaborative support network. Future research may focus on the development of a comprehensive nursing care model encompassing “physiological intervention, technical guidance, psychological counseling, and family support” to effectively alleviate breastfeeding barriers in the early postpartum period.

## 6 Limitations and future directions

This study has several limitations. First, its cross-sectional design only allows for the identification of statistical associations between variables and breastfeeding difficulties in the early postpartum period, precluding the inference of definitive causal relationships. Second, the use of single-center convenience sampling, involving only one tertiary specialized hospital in Chengdu, may limit the generalizability of the findings, given the potential variations in clinical support and health education for breastfeeding across different regions and levels of healthcare facilities. Third, the assessment of study variables relied primarily on observational scales and self-reported questionnaires, without integrating objective biological markers, such as prolactin, progesterone, and oxytocin, to provide multidimensional cross-validation. Fourth, the directionality between elevated postpartum depressive symptoms and breastfeeding difficulties cannot be definitively determined. While depressive symptoms may negatively impact lactation, the unexpected barriers and failure to achieve initial breastfeeding goals can conversely precipitate poor maternal mental health. Fifth, while we evaluated maternal factors and general latching success, we did not comprehensively assess the infant's specific oral motor skills or milk removal efficiency. As breastfeeding is a dynamic dyadic process, the maternal side represents only half of the picture. Furthermore, various birth interventions may directly affect the infant's neurological and oral abilities to latch and nurse effectively, which in turn impacts early milk supply. This highlights a significant gap, and future lactation physiology studies should incorporate specialized neonatal oral skill assessments to provide a holistic evaluation.

In light of these limitations, future research should prioritize multi-center, large-scale prospective cohort studies to further clarify the temporal relationships of these associated factors. Additionally, it is recommended that future studies incorporate objective endocrinological indicators to explore the physiological mechanisms underlying delayed secretory activation from the perspective of hormone metabolism. Furthermore, we look forward to future nursing research that utilizes the core associated factors identified in this study to develop systematic and personalized clinical intervention protocols. Finally, the efficacy of these interventions should be validated through randomized controlled trials to provide higher-level evidence for optimizing breastfeeding support pathways in Baby-Friendly Hospitals.

## Data Availability

The raw data supporting the conclusions of this article will be made available by the authors, without undue reservation.

## References

[1] UNICEF. 2025 Nutrition for Growth Summit: Four SMART Breastfeeding Commitments; Ten Steps to Successful Breastfeeding. New York, NY: UNICEF (2025).

[2] World health Organization. Global Nutrition Targets 2030: Breastfeeding Brief. Geneva: World health Organization (2025).

[3] Black RE Victora CG Walker SP Bhutta ZA Christian P de Onis M . Maternal and child undernutrition and overweight in low-income and middle-income countries. Lancet. (2013) 382:427–51. doi: 10.1016/S0140-6736(13)60937-X 23746772

[4] Victora CG Bahl R Barros AJ França GV Horton S Krasevec J . Breastfeeding in the 21st century: epidemiology, mechanisms, and lifelong effect. Lancet. (2016) 387:475–90. doi: 10.1016/S0140-6736(15)01024-7 26869575

[5] Meek JY Noble L. Policy statement: breastfeeding and the use of human milk. Pediatrics. (2022) 150:2022057988. doi: 10.1542/peds.2022-057988 35921640

[6] Aune D Norat T Romundstad P Vatten LJ. Breastfeeding and the maternal risk of type 2 diabetes: a systematic review and dose-response meta-analysis of cohort studies. Nutr Metab Cardiovasc Dis. 24:107–115. doi: 10.1016/j.numecd.2013.10.028 24439841

[7] Chen Y Jiang P Geng Y. The role of breastfeeding in breast cancer prevention: a literature review. Front Oncol. (2023) 13:1257804. doi: 10.3389/fonc.2023.1257804 PMC10512942 37746260

[8] Obeagu EI Obeagu GU. Exploring the profound link: Breastfeeding's impact on alleviating the burden of breast cancer - a review. Medicine. (2024) 103:e37695. doi: 10.1097/MD.0000000000037695 PMC11018178 38608095

[9] Group Group of Child Health Care the the Society of Pediatrics Chinese Medical Association Group Group of Clinical Nutrition the the Society of Pediatrics Chinese Medical Association Society Society of Perinatal Medicine Chinese Medical Association Chinese Nutrition Society Maternal and Child Nutrition Branch and and Editorial Board of the Chinese Journal of Pediatrics. Strategies for breastfeeding promotion guidelines. Chin J Pediatr. (2026) 64:467–78.

[10] Chen C Yan Y Gao X Xiang S He Q Zeng G . Influences of cesarean delivery on breastfeeding practices and duration: a prospective cohort study. J Hum Lact. (2018) 34:526–34. doi: 10.1177/0890334417741434 29365288

[11] Özer Aslan I Aslan MT Can N Sevinç Ergül Ö Çallioglu N. Impact of delivery method on initiation and continuation of breastfeeding: a prospective cohort study. Children. (2025) 12:966. doi: 10.3390/children12080966 PMC12384195 40868418

[12] Lian W Ding J Xiong T Liuding J Nie L. Determinants of delayed onset of lactogenesis II among women who delivered via Cesarean section at a tertiary hospital in China: a prospective cohort study. Int Breastfeed J. (2022) 17:81. doi: 10.1186/s13006-022-00523-3 PMC9714018 36451171

[13] McManaman JL Neville MC. Mammary physiology and milk secretion. Adv Drug Deliv Rev. (2003) 55:629–41. doi: 10.1016/S0169-409X(03)00033-4 12706546

[14] Grattan RE London SM Bueno GE. Perceived pressure to breastfeed negatively impacts postpartum mental health outcomes over time. Front Public Health. (2024) 12:1357965. doi: 10.3389/fpubh.2024.1357965 PMC11024305 38638486

[15] Parker LA Sullivan S Krueger C Kelechi T Mueller M. Effect of early breast milk expression on milk volume and timing of lactogenesis stage II among mothers of very low birth weight infants: a pilot study. J Perinatol. (2012) 32:205–9. doi: 10.1038/jp.2011.78 21904296

[16] Dunn RL Kalich KA Fedrizzi R Phillips S. Barriers and contributors to breastfeeding in WIC mothers: a social ecological perspective. Breastfeed Med. (2015) 10:493–501. doi: 10.1089/bfm.2015.0084 26565749

[17] Demirci JR Bogen DL. An ecological momentary assessment of primiparous women's breastfeeding behavior and problems from birth to 8 weeks. J Hum Lact. (2017) 33:285–95. doi: 10.1177/0890334417695206 PMC5512283 28418803

[18] Medina Poeliniz C Engstrom JL Hoban R Patel AL Meier P. Measures of secretory activation for research and practice: an integrative review. Breastfeed Med. (2020) 15:191–212. doi: 10.1089/bfm.2019.0247 32155345

[19] Shahrani ASA Hushan HM Binjamaan NK Binhuwaimel WA Alotaibi JJ Alrasheed LA. Factors associated with early cessation of exclusive breast feeding among Saudi mothers: a prospective observational study. J Family Med Prim Care. (2021) 10:3657–63. doi: 10.4103/jfmpc.jfmpc_852_21 PMC8653446 34934662

[20] Rollins NC Bhandari N Hajeebhoy N Horton S Lutter CK Martines JC . Why invest, and what it will take to improve breastfeeding practices? Lancet. (2016) 387:491–504. doi: 10.1016/S0140-6736(15)01044-2 26869576

[21] Peng Y Zhuang K Huang Y. Incidence and factors influencing delayed onset of lactation: a systematic review and meta-analysis. Int Breastfeeding J. (2024) 19:59. doi: 10.1186/s13006-024-00666-5 PMC11342634 39175092

[22] Peduzzi P Concato J Kemper E Holford TR Feinstein AR. A simulation study of the number of events per variable in logistic regression analysis. J Clin Epidemiol. (1996) 49:1373–9. doi: 10.1016/S0895-4356(96)00236-3 8970487

[23] Jensen D Wallace S Kelsay P. LATCH: a breastfeeding charting system and documentation tool. J Obstet Gynecol Neonatal Nurs. (1994) 23:27–32. doi: 10.1111/j.1552-6909.1994.tb01847.x 8176525

[24] Ding LS Wang Y Luo S. Effect of kangaroo mother care on breastfeeding and behavioral status of full-term newborns. Chin J Pract Nurs. (2018) 34:1877–82. doi: 10.1111/jog.16132

[25] Dennis CL. The breastfeeding self-efficacy scale: psychometric assessment of the short form. J Obstet Gynecol Neonatal Nurs. (2003) 32:734–44. doi: 10.1177/0884217503258459 14649593

[26] Liu YW Dong X. Reliability and validity of the Chinese version of Breastfeeding self-efficacy scale-short form. Chin J Pract Nurs. (2016) 32:1361–4. doi: 10.3760/cma.j.issn.1672-7088.2016.18.001

[27] Cox JL Holden JM Sagovsky R. Detection of postnatal depression. Development of the 10-item Edinburgh postnatal depression scale. Br J Psychiatry. (1987) 150:782–6. doi: 10.1192/bjp.150.6.782 3651732

[28] Wang YQ Guo XJ. Cut-off value of Edinburgh postnatal depression scale for screening postpartum depression among pregnant women in Chengdu. Chin J Pract Nurs. (2009) 25:1–4.

[29] Boss M Gardner H Hartmann P. Normal human lactation: closing the gap. F1000Res. (2018) 7:F100. doi: 10.12688/f1000research.14452.1 PMC6013763 29983914

[30] Bengough T Dawson S Cheng HL McFadden A Gavine A Rees R . Factors that influence women's engagement with breastfeeding support: a qualitative evidence synthesis. Matern Child Nutr. (2022) 18:e13405. doi: 10.1111/mcn.13405 PMC9480951 36006012

[31] Nommsen-Rivers LA. Does insulin explain the relation between maternal obesity and poor lactation outcomes? An overview of the literature. Adv Nutr. (2016) 7:407–14. doi: 10.3945/an.115.011007 PMC4785481 26980825

[32] Cuffe C Giglia R Hill J Cooper MN Moorhead AM Lewis JR . Risk factors associated with delayed secretory activation in a cohort of nulliparous women in Western Australia. J Hum Lact. (2026) 1–11. doi: 10.1177/08903344261435721. [Epub ahead of print]. 42046466

[33] Chow KM Leung AWY Hui LL Yeung KHT Lau EYY Rollins NC . Factors associated with exclusive breastfeeding at 6 months postpartum: a prospective mixed-method cohort study. Pediatr Res. (2026). doi: 10.1038/s41390-025-04731-5. [Epub ahead of print]. 41501414

[34] Gianni ML Bettinelli ME Manfra P Sorrentino G Bezze E Plevani L . Breastfeeding difficulties and risk for early breastfeeding cessation. Nutrients. (2019) 11:2266. doi: 10.3390/nu11102266 PMC6835226 31547061

[35] Uvnäs Moberg K Ekström-Bergström A Buckley S Massarotti C Pajalic Z Luegmair K . Maternal plasma levels of oxytocin during breastfeeding-a systematic review. PLoS ONE. (2020) 15:e0235806. doi: 10.1371/journal.pone.0235806 PMC7406087 32756565

[36] Pérez-Escamilla R Tomori C Hernández-Cordero S Baker P Barros AJD Bégin F . Breastfeeding: crucially important, but increasingly challenged in a market-driven world. Lancet. (2023) 401:472–85. doi: 10.1016/S0140-6736(22)01932-8 36764313

[37] Wu JL Pang SQ Jiang XM Zheng QX Han XQ Zhang XY . Gestational diabetes mellitus and risk of delayed onset of lactogenesis: a systematic review and meta-analysis. Breastfeed Med. (2021) 16:385–92. doi: 10.1089/bfm.2020.0356 33891507

[38] Horsley K Chaput K Da Costa D Nguyen TV Dayan N Tomfohr-Madsen L . Hypertensive disorders of pregnancy and breastfeeding practices: A secondary analysis of data from the All Our Families Cohort. Acta Obstet Gynecol Scand. (2022) 101:871–9. doi: 10.1111/aogs.14378 PMC9564688 35610941

[39] Flaherman VJ Gay B Scott C Avins A Lee KA Newman TB. Randomised trial comparing hand expression with breast pumping for mothers of term newborns feeding poorly. Arch Dis Child Fetal Neonatal Ed. (2012) 97:F18–23. doi: 10.1136/adc.2010.209213 PMC5332145 21747129

[40] Lawrence RA Lawrence RM. 3 - Physiology of lactation. In: Breastfeeding. 9th edn. Philadelphia: Elsevier (2022), *p*. 58–92. doi: 10.1016/B978-0-323-68013-4.00003-1

[41] Lin SY Lee JT Yang CC Gau ML. Factors related to milk supply perception in women who underwent cesarean section. J Nurs Res. (2011) 19:94–101. doi: 10.1097/JNR.0b013e31821988e9 21586986

[42] Brugaletta C Le Roch K Saxton J Bizouerne C McGrath M Kerac M. Breastfeeding assessment tools for at-risk and malnourished infants aged under 6 months old: a systematic review. F1000Res. (2020) 9:1310. doi: 10.12688/f1000research.24516.1 PMC7898355 33628437

[43] McClellan HL Hepworth AR Garbin CP Rowan MK Deacon J Hartmann PE. Nipple pain during breastfeeding with or without visible trauma. J Hum Lact. (2012) 28:511–21. doi: 10.1177/0890334412444464 22689707

[44] Moore ER Anderson GC Bergman N Dowswell T. Early skin-to-skin contact for mothers and their healthy newborn infants. Cochrane Database Syst Rev. (2012) 5:Cd003519. doi: 10.1002/14651858.CD003519.pub3 PMC3979156 22592691

[45] Bruce KE Wouk K Grewen KM Pearson B Meltzer-Brody S Stuebe AM . HPA axis dysregulation and postpartum depression and anxiety symptoms in breastfeeding vs bottle-feeding parents. Psychoneuroendocrinology. (2025) 172:107253. doi: 10.1016/j.psyneuen.2024.107253 PMC11830542 39675161

[46] Brockway M Benzies K Hayden KA. Interventions to improve breastfeeding self-efficacy and resultant breastfeeding rates: a systematic review and meta-analysis. J Hum Lact. (2017) 33:486–99. doi: 10.1177/0890334417707957 28644764

[47] Shorey S Chan SW Chong YS He HG. Perceptions of primiparas on a postnatal psychoeducation programme: the process evaluation. Midwifery. (2015) 31:155–63. doi: 10.1016/j.midw.2014.08.001 25174540

