# Supplementary material for: Factors associated with breastfeeding difficulties in the early postpartum period in China: a cross-sectional study
Source: Front Public Health. 2026 Oct 20;14:1923736. doi: 10.3389/fpubh.2026.1923736 (PMC13727518; doi:10.3389/fpubh.2026.1923736)
Supplement: Supplementary file 1 [file Supplementary_File_1.pdf]

Supplementary Table S1. Detailed clinical observations of maternal breast and neonatal status associated with breastfeeding difficulties in the early postpartum period

Characteristics	Without breastfeeding difficulties（n=283）	With breastfeeding difficulties（n=177）	Test statistic	P
Degree of breast pain			1.985	0.576
No pain	121 (42.76%)	79 (44.63%)		
Mild	78 (27.56%)	47 (26.55%)		
Moderate	59 (20.85%)	30 (16.95%)		
Severe	25 (8.83%)	21 (11.86%)		
Nipple examination			0.847	0.357
No abnormality	180 (63.6%)	105 (59.32%)		
Abnormality present	103 (36.4%)	72 (40.68%)		
Areola examination			0.004	0.947
No abnormality	175 (61.84%)	110 (62.15%)		
Abnormality present	108 (38.16%)	67 (37.85%)		
Location of pain			3.995	0.136
Superficial	106 (37.46%)	65 (36.72%)		
Deep	123 (43.46%)	65 (36.72%)		
Both	54 (19.08%)	47 (26.55%)		
Duration of pain			2.904	0.234
During breastfeeding	172 (60.78%)	115 (64.97%)		
Not during breastfeeding	57 (20.14%)	39 (22.03%)		
Both	54 (19.08%)	23 (12.99%)		
Breast engorgement			6.269	0.099

No	159 (56.18%)	83 (46.89%)		
Mild (as soft as lips)	71 (25.09%)	49 (27.68%)		
Moderate (as firm as the nose tip)	36 (12.72%)	36 (20.34%)		
Severe (as hard as the forehead)	17 (6.01%)	9 (5.08%)		
Breast lump/mass			4.418	0.11
NO	221 (78.09%)	152 (85.88%)		
Unilateral	50 (17.67%)	21 (11.86%)		
Bilateral	12 (4.24%)	4 (2.26%)		
Breast scar			Fisher	0.259
NO	255 (90.11%)	161 (90.96%)		
Unilateral	26 (9.19%)	12 (6.78%)		
Bilateral	2 (0.71%)	4 (2.26%)		
Mastitis infection			4.261	0.119
NO	266 (93.99%)	157 (88.7%)		
Unilateral	12 (4.24%)	13 (7.34%)		
Bilateral	5 (1.77%)	7 (3.95%)		
Milk secretion volume			0.672	0.88
No	66 (23.32%)	47 (26.55%)		
Moderate volume	146 (51.59%)	88 (49.72%)		
High volume	71 (25.09%)	42 (23.73%)		
Apgar score			Fisher	0.867
0-3 points	5 (1.77%)	3 (1.69%)		
4-7 points	8 (2.83%)	7 (3.95%)		
8-10 points	270 (95.41%)	167 (94.35%)		
Mental status			3.051	0.549

Alert	103 (36.4%)	67 (37.85%)		
Easily comforted	78 (27.56%)	58 (32.77%)		
Difficult to comfort	29 (10.25%)	16 (9.04%)		
Somnolent and easily aroused	50 (17.67%)	27 (15.25%)		
Somnolent and difficult to arouse	23 (8.13%)	9 (5.08%)		
Lingual frenulum			5.881	0.053
Normal	259 (91.52%)	149 (84.18%)		
Short	14 (4.95%)	17 (9.6%)		
Not examined	10 (3.53%)	11 (6.21%)		
Cleft lip/palate			0	1
Normal	282 (99.65%)	176 (99.44%)		
Cleft lip/Cleft palate	1 (0.35%)	1 (0.56%)		
Oral thrush			0.579	0.447
Present	13 (4.59%)	11 (6.21%)		
Absent	270 (95.41%)	166 (93.79%)		
Other neonatal abnormalities			Fisher	0.995
No	223 (78.8%)	143 (80.79%)		
Jaundice	26 (9.19%)	16 (9.04%)		
Pneumonia	8 (2.83%)	4 (2.26%)		
Rash	14 (4.95)	8 (4.52%)		
Diarrhea	7 (2.47)	3 (1.69%)		
Allergy	4 (1.41%)	3 (1.69%)		
Other	1 (0.35%)	0 (0%)		
Feeding method			3.001	0.223
Exclusive breastfeeding	187 (66.08%)	109 (61.58%)		

Mixed feeding	70 (24.73%)	56 (31.64%)		
Artificial feeding	26 (9.19%)	12 (6.78%)		
Rooting reflex			2.858	0.826
Active rooting/mouth opening	79 (27.92%)	52 (29.38%)		
Difficulty in eliciting rooting	28 (9.89%)	14 (7.91%)		
Spontaneous wide mouth opening	71 (25.09%)	40 (22.6%)		
Wide mouth opening after stimulation	54 (19.08%)	36 (20.34%)		
Getting upset at the breast	12 (4.24%)	11 (6.21%)		
Breast refusal	11 (3.89%)	4 (2.26%)		
No response	28 (9.89%)	20 (11.3%)		
Latch status			6.049	0.534
Proper latch: breast held, tongue down, lips flanged, rhythmic sucking	35 (12.37%)	23 (12.99)		
Repeated attempts	39 (13.78%)	20 (11.3%)		
Nipple-only latch	32 (11.31%)	21 (11.86%)		
No latch	42 (14.84%)	21 (11.86%)		
Lower lip turned inward	27 (9.54%)	25 (14.12%)		
Upper lip turned inward	44 (15.55%)	20 (11.3%)		
Unable to maintain latch, slips off easily	32 (11.31%)	27 (15.25%)		
Other	32 (11.31%)	20 (11.3%)		
Sucking/swallowing status			6.074	0.415

Swallowing present	32 (11.31%)	24 (13.56%)		
Sucking requires stimulation	40 (14.13%)	22 (12.43%)		
Weak sucking	54 (19.08%)	24 (13.56%)		
10–20 bursts of rapid sucking	39 (13.78%)	25 (14.12%)		
Pauses followed by spontaneous resumption after 3–5 seconds	45 (15.9%)	24 (13.56%)		
Intermittent/discontinuous	31 (10.95%)	30 (16.95%)		
The nipple slid out during 1–3 sucks	42 (14.84%)	28 (15.82%)		
Maternal response during latching			3.371	0.338
No	148 (52.3%)	77 (43.5%)		
Mild	81 (28.62%)	60 (33.9%)		
Moderate	42 (14.84%)	31 (17.51%)		
Severe	12 (4.24%)	9 (5.08%)		
Breastfeeding position			0.553	0.907
Cradle hold	136 (48.06%)	90 (50.85%)		
Side-lying position	70 (24.73%)	40 (22.6%)		
Football hold	47 (16.61%)	27 (15.25%)		
Cross-cradle hold	30 (10.6%)	20 (11.3%)		
Passing flatus			1.674	0.196
No	62 (21.91%)	30 (16.95%)		
Yes	221 (78.09%)	147 (83.05%)		
Presence of abdominal distension			1.037	0.308

No	247 (87.28%)	160 (90.4%)		
Yes	36 (12.72%)	17 (9.6%)		
Diet			3.175	0.365
Liquid diet	21 (7.42%)	19 (10.73%)		
Semi-liquid diet	55 (19.43%)	37 (20.9%)		
Soft diet	106 (37.46%)	70 (39.55%)		
Regular diet	101 (35.69%)	51 (28.81%)		
Primary caregiver			0.86	0.835
No	59 (20.85%)	32 (18.08%)		
Husband	41 (14.49%)	26 (14.69%)		
Mother	145 (51.24%)	91 (51.41%)		
Caregiver	38 (13.43%)	28 (15.82%)		

Note: Test statistics represent Z values derived from the Mann-Whitney U test for non-normally distributed continuous variables, and X^2 values for categorical variables, unless explicitly marked as Fisher's exact test. For categorical variables with more than two levels, the reported P value represents the overall test across all categories.

Supplementary Table S2. Sensitivity Analysis: Multivariable logistic regression model excluding the LATCH score

Variables	B	SE	Wald X^2	p value	OR (95% CI)
(Intercept)	1.740	0.984	3.122	0.077	5.695(0.827-39.203)
Breastfeeding self-efficacy	-0.035	0.016	4.687	0.030	0.966(0.936-0.997)
Family support	-0.088	0.027	10.317	0.001	0.916(0.868-0.966)
Age					
< 35 years					1.00 (Ref.)
≥ 35 years	0.456	0.232	3.849	0.050	1.578(1.001-2.487)
Mode of delivery					
Vaginal delivery					1.00 (Ref.)

Cesarean section	0.511	0.227	5.049	0.025	1.667(1.068-2.603)
Parity					
1 time					1.00 (Ref.)
2 times	-0.940	0.365	6.625	0.010	0.391(0.191-0.799)
≥ 3 times	-0.888	0.480	3.430	0.064	0.411(0.161-1.053)
Daily breastfeeding frequency					
8-12 times/day					1.00 (Ref.)
< 6 times/day	1.020	0.240	18.079	<0.001	2.774(1.733-4.439)
6-7 times/day	-0.091	0.740	0.015	0.902	0.913(0.214-3.891)
Hypertensive disorders of pregnancy					
No					1.00 (Ref.)
Yes	0.763	0.299	6.518	0.011	2.144(1.194-3.851)
Gestational Diabetes Mellitus					
No					1.00 (Ref.)
Yes	0.636	0.259	6.042	0.014	1.889(1.138-3.137)
BMI classification					
< 18.5					1.00 (Ref.)
18.5-23.9	0.338	0.315	1.156	0.282	1.402(0.757-2.599)
24-27.9	1.010	0.394	6.569	0.010	2.746(1.268-5.944)
≥28	1.049	0.696	2.271	0.132	2.855(0.729-11.173)
Elevated depressive symptoms					
No					1.00 (Ref.)
Yes	1.331	0.364	13.381	<0.001	3.783 (1.855-7.719)

Supplementary File 2. Structured Clinical Breastfeeding Assessment Checklist

Participant ID: ________________________

Assessor ID (IBCLC Certified Nurse): ________________________

Exact Time of Assessment: ______ Hours Postpartum (Must be between 24 and 96 hours)

Date of Assessment (DD/MM/YYYY): ____/____/________

General Instructions for the Assessor:

This checklist must be completed during an active breastfeeding session at the mother's bedside. Please conduct a direct physical examination and structured observation. Do not rely solely on maternal self-report for these clinical domains.

Domain 1: Maternal Physiological Assessment

1.1 Nipple Examination (Check all that apply)

Left: ☐Normal ☐Flat ☐Inverted ☐Enlarged ☐Edema ☐Fissure ☐Engorgement ☐Pain ☐Deformed ☐Bleeding ☐Scab ☐White spot/Blister

Right: ☐Normal ☐Flat ☐Inverted ☐Enlarged ☐Edema ☐Fissure ☐Engorgement ☐Pain ☐Deformed ☐Bleeding ☐Scab ☐White spot/Blister

1.2 Areola Examination (Check all that apply)

Left: ☐Normal ☐Small ☐Large ☐Edema ☐Engorgement ☐Pain ☐Bleeding ☐Scab ☐Blister☐☐

Right: ☐Normal ☐Small ☐Large ☐Edema ☐Engorgement ☐Pain ☐Bleeding ☐Scab ☐Blister

1.3 Breast Pain Assessment

Degree of pain: ☐None ☐Mild ☐Moderate ☐Severe

Location of pain: ☐Superficial ☐Deep ☐Both

Duration/Timing of pain: ☐During breastfeeding ☐Not during breastfeeding ☐Both

1.4 Breast Physical Examination

Breast engorgement severity:

☐None

☐Mild (Grade I, as soft as lips)

☐Moderate (Grade II, as firm as the nose tip)

☐Severe (Grade III, as hard as the forehead)

Breast mass/lump: □None □Unilateral □Bilateral

Breast scar: □None □Unilateral □Bilateral

Mastitis infection: □None □Unilateral □Bilateral

Domain 2: Neonatal Status Assessment

2.1 Neonatal Vitality & Behavior

Mental status prior to feeding: □Alert □Easily comforted □Difficult to comfort □Somnolent and easily aroused □Somnolent and difficult to arouse

2.2 Neonatal Oral Examination

Lingual frenulum: □Normal □Short □Not examined

Congenital/Oral anomalies:

Cleft lip / Cleft palate: □ Present □ Absent

Oral thrush: □ Present □ Absent

Other acute neonatal abnormalities: □None □Jaundice □Pneumonia □Rash □Diarrhea □Allergy

Domain 3: Breastfeeding Process Assessment

3.1 Infant Rooting and Approach

Rooting reflex performance:

□Active rooting/mouth opening

□Difficulty in eliciting rooting

□Spontaneous wide mouth opening

□Wide mouth opening after stimulation

□Getting upset at the breast

□Breast refusal

□No response

3.2 Infant Latching Status

Latch quality (Identify the primary status):

□Proper latch (breast held, tongue down, lips flanged, rhythmic sucking)

□Repeated attempts

☐Nipple-only latch

☐No latch

☐Lower lip turned inward

☐Upper lip turned inward

☐Unable to maintain latch, slips off easily

☐Other

3.3 Sucking and Swallowing Patterns

Sucking efficacy and continuity:

☐Swallowing present

☐Sucking requires stimulation

☐Weak sucking

☐10–20 bursts of rapid sucking

☐Pauses followed by spontaneous resumption after 3–5 seconds

☐Intermittent/discontinuous

☐The nipple slid out during 1–3 sucks

3.4 Maternal Response

Maternal pain response during active latching:

☐None

☐Mild

☐Moderate

☐Severe

Domain 4: Maternal Nutritional Status Assessment

4.1 Postpartum Diet Status

Current dietary intake: ☐Liquid diet ☐Semi-liquid diet ☐Soft diet ☐Regular diet

[CLINICAL CLASSIFICATION ALGORITHM]

Based on the structured assessment above, does the mother-infant dyad exhibit Early Postpartum Breastfeeding Difficulties?

☐YES

□NO

Diagnostic Criteria for "YES": Ensure at least ONE of the following is documented during this assessment:

(1) Perceived insufficient milk supply causing significant maternal anxiety or leading to non-medically indicated formula supplementation.

(2) Nipple or breast-related problems (e.g., severe pain disrupting feeding, visible nipple trauma, or severe breast engorgement).

(3) Neonatal or interactive factors physically impeding effective feeding (e.g., infant inability to maintain a latch, severe crying at the breast).

Assessor Signature: ____________________ Date: _________________________
